# Risk of Relapse Post Reduced Intensity Conditioning Allogeneic Stem Cell Transplant in Patients With High‐Risk Myeloid Neoplasms Based on GvHD Prophylaxis: PTCy Vs. TAC/MTX

**DOI:** 10.1002/ajh.70059

**Published:** 2025-09-04

**Authors:** Khalil Hassan, Anmol Baranwal, Al Rabee Kassis, Jade Braun, Gabriel Bartoo, Robert Wolf, Mehrdad Hefazi, Aasiya Matin, Abhishek Mangoankar, Mithun V. Shah, Mark R. Litzow, William J. Hogan, David Dingli, Hassan B. Alkhateeb

**Affiliations:** ^1^ Division of Hematology Mayo Clinic Rochester Rochester Minnesota USA; ^2^ Cancer Centers of Southwest Oklahoma Lawton Oklahoma USA; ^3^ Department of Pharmacy Mayo Clinic Rochester Minnesota USA

**Keywords:** allogeneic transplant, AML, CMML, MDS, post‐transplant cyclophosphamide

To the Editor,

1

The recent CTN 1703 clinical trial has paved the way for post‐transplant cyclophosphamide with tacrolimus and mycophenolate (PTCy) to become the new standard of care for graft‐versus‐host disease (GvHD) prophylaxis in patients undergoing matched related donor (MRD) and matched unrelated donor (MUD) allogeneic hematopoietic stem cell transplantation (alloHCT) using reduced intensity conditioning [1]. In the parent trial, PTCy was associated with reduced incidence of grade 3–4 acute GvHD and chronic GvHD compared to tacrolimus/methotrexate (TAC/MTX) without significantly affecting relapse risk or overall survival outcomes [1]. Recently Holtan et al. reported the 2‐year outcomes of the CTN 1703 trial, confirming superior graft‐versus‐host disease‐free, relapse‐free survival (GRFS) in the PTCy arm compared to the TAC/MTX arm (42.4% vs. 28.8%, *p* = 0.001) [2]. Notably, there were no significant differences in disease‐free survival (DFS), overall survival (OS), relapse, or non‐relapse mortality between the two groups [2].

Approximately one‐third of patients in both arms had high/very high disease risk index (DRI). Since no prior studies have compared the risk of relapse between PTCy and TAC/MTX‐based GvHD prophylaxis in this population, our study aimed to evaluate the relapse risk in patients with high‐risk myeloid neoplasms (HR‐MNs) undergoing reduced‐intensity (RIC) or non‐myeloablative (NMA) conditioning alloHCT while receiving either PTCy or TAC/MTX for GvHD prophylaxis.

We conducted a retrospective analysis of patients with HR‐MNs who underwent RIC/NMA alloHCT between January 2018 and June 2023 at Mayo Clinic Rochester. Criteria for HR‐MN included acute myeloid leukemia (AML) with ≥ 1 adverse feature (complex (CK) or monosomal karyotype (MK), *TP53* mutation, *WT1* mutation, *FLT3 ITD+/NPM1*‐, active disease, positive flow cytometric or molecular minimal residual disease (MRD+), or secondary AML), myelodysplastic syndrome with ≥ 1 risk factor (CK/MK, *TP53* mutation, *RAS* pathway mutation, marrow blasts ≥ 10%), or chronic myelomonocytic leukemia [3, 4, 5]. Statistical analyses included Kaplan–Meier estimates and log‐rank tests to assess OS and DFS, as well as competing risk analysis to calculate non‐relapse mortality (NRM) and cumulative incidence of relapse (CIR). Multivariate analysis was used to evaluate independent predictors of relapse, adjusting for covariates identified in univariate analysis. Collinearity among variables in multivariate analysis was assessed by evaluation for variance inflation factor (VIF). VIF of 5 or above was considered high and suggestive of collinearity.

Out of 552 alloHCTs, 107 consecutive patients (19.3%) met the inclusion criteria. Of these, 71 patients (66.3%) received TAC/MTX, while 36 (33.6%) received PTCy. The median follow‐up was shorter in the PTCy group compared to the TAC/MTX group (19 months vs. 46 months, *p* < 0.0001). The median age at transplant across both cohorts was 65 years (range: 31–76 years), with no significant difference between the two groups (*p* = 0.59) (Table 1).

**TABLE 1:** Characteristics and transplant outcomes of patients receiving post‐transplant cyclophosphamide vs. tacrolimus/methotrexate.

Variable	PTCY based (*N* = 36)	TAC, MTX (*N* = 71)	*p*
Age at diagnosis			0.51
Median (Q1, Q3)	63.0 (60.5, 66.5)	64.0 (62.0, 67.5)	
Age at transplant			0.59
Median (Q1, Q3)	64.0 (61.8, 68.0)	65.0 (63.5, 69.0)	
Donor source			< 0.01
Haplo	12 (33.3%)	0 (0.0%)	
MMUD	0 (0.0%)	2 (2.8%)	
MRD	1 (2.8%)	19 (26.8%)	
MUD	23 (63.9%)	50 (70.4%)	
Disease			0.44
AML	21 (58.3%)	36 (50.7%)	
CMML	4 (11.1%)	15 (21.1%)	
MDS	11 (30.6%)	20 (28.2%)	
Molecular mutations			
*TP53*	9 (25.0%)	13 (18.3%)	0.42
Multi‐hit (per ICC)	7 (19.4%)	11 (15.5%)	0.61
*WT1*	4 (11.1%)	3 (4.2%)	0.17
*ASXL1*	9 (25.0%)	21 (29.6%)	0.62
*BCOR*	2 (5.6%)	6 (8.5%)	0.59
*CEBPA*	3 (8.3%)	2 (2.8%)	0.20
*FLT3*	2 (5.6%)	9 (12.7%)	0.25
*IDH1*	2 (5.6%)	3 (4.2%)	0.76
*IDH2*	2 (5.6%)	9 (12.7%)	0.25
*GATA2*	2 (5.6%)	2 (2.8%)	0.48
*JAK2*	1 (2.8%)	2 (2.8%)	0.99
*KRAS*	2 (5.6%)	2 (2.8%)	0.48
*NRAS*	6 (16.7%)	7 (9.9%)	0.31
*PHF6*	2 (5.6%)	2 (2.8%)	0.48
*RUNX1*	8 (22.2%)	13 (18.3%)	0.63
Cytogenetic abnormalities			
Monosomal karyotype			0.99
No	35 (97.2%)	69 (97.2%)	
Yes	1 (2.8%)	2 (2.8%)	
Complex karyotype			0.69
No	24 (66.7%)	50 (70.4%)	
Yes	12 (33.3%)	21 (29.6%)	
CK/MK			0.39
No	30 (83.3%)	54 (76.1%)	
Yes	6 (16.7%)	17 (23.9%)	
Chromosome 17 cytogenetic abnormality			0.98
No	31 (86.1%)	61 (85.9%)	
Yes	5 (13.9%)	10 (14.1%)	
Chromosome 7 cytogenetic abnormality			0.26
No	26 (72.2%)	58 (81.7%)	
Yes	10 (27.8%)	13 (18.3%)	
Secondary AML			0.89
No	29 (80.6%)	58 (81.7%)	
Yes	7 (19.4%)	13 (18.3%)	
ELN 2022			0.66
Adverse	15 (71.4%)	27 (75.0%)	
Intermediate	2 (9.5%)	5 (13.9%)	
Favorable	4 (19.0%)	4 (11.1%)	
IPSS‐R at diagnosis			0.69
High or Very High	8 (80.0%)	13 (68.4%)	
Intermediate	2 (20.0%)	5 (26.3%)	
Low	0 (0.0%)	1 (5.3%)	
Disease status pre‐transplant			0.03
Active disease	4 (11.1%)	21 (29.6%)	
CR	32 (88.9%)	50 (70.4%)	
BM BLASTS % ≥ 5 PRE‐TRANSPLANT			0.26
No	35 (97.2%)	65 (91.5%)	
Yes	1 (2.8%)	6 (8.5%)	
AML MRD			0.12
Negative	11 (55.0%)	21 (80.8%)	
Positive	9 (45.0%)	5 (19.2%)	
High or Very high DRI			0.30
No	21 (67.7%)	31 (56.4%)	
Yes	10 (32.3%)	24 (43.6%)	
KPS ≥ 80			0.15
No	5 (13.9%)	4 (5.6%)	
Yes	31 (86.1%)	67 (94.4%)	
HCT‐CI ≥ 3			0.09
No	22 (61.1%)	31 (43.7%)	
Yes	14 (38.9%)	40 (56.3%)	
Conditioning regimen			0.05
Cy/Flu/TBI	2 (5.6%)	0 (0.0%)	
Flu/Bu/Thiotepa	1 (2.8%)	0 (0.0%)	
Flu/Mel+/‐TBI	33 (91.7%)	71 (100.0%)	
aGVHD II‐IV			0.24
No	30 (83.3%)	52 (73.2%)	
Yes	6 (16.7%)	19 (26.8%)	
aGVHD III‐IV			0.15
No	32 (88.9%)	55 (77.5%)	
Yes	4 (11.1%)	16 (22.5%)	
cGVHD moderate/severe.			< 0.01
No	28 (77.8%)	36 (50.7%)	
Yes	8 (22.2%)	35 (49.3%)	
Post transplant maintenance			0.59
No	23 (63.9%)	49 (69.0%)	
Yes	13 (36.1%)	22 (31.0%)	
Relapse after transplant			< 0.01
No	20 (55.6%)	58 (81.7%)	
Yes	16 (44.4%)	13 (18.3%)	
Alive/Dead			0.84
Alive	20 (55.6%)	38 (53.5%)	
Dead	16 (44.4%)	33 (46.5%)	

Abbreviations: aGVHD, acute graft‐versus‐host disease; AML, acute myeloid leukemia; BM, bone marrow; cGVHD, chronic graft‐versus‐host disease; CMML, chronic myelomonocytic leukemia; CR, complete remission; Cy, cyclophosphamide; DRI, disease risk index; Flu, fludarabine; Haplo, haploidentical; HCT‐CI, hematopoietic cell transplantation‐specific comorbidity index; IPSS‐R, revised International Prognostic Scoring System; KPS, Karnofsky performance status; MDS, Myelodysplastic syndrome; Mel, Melphalan; MMUD, mismatched unrelated donor; MRD, matched related donor; MRD, minimal residual disease; MUD, matched unrelated donor; TBI, total body irradiation.

In the PTCy cohort, 12 (33.3%) received haploidentical transplants, whereas the remaining 24 (66.7%) received MRD or MUD transplants. Conversely, within the TAC/MTX group, 69 (97.2%) of patients received MUD or MRD transplants, and 2 (2.8%) received mismatched unrelated transplants (MMUD). The distribution of underlying diseases was comparable between the PTCy and TAC/MTX groups: AML (58.3% vs. 50.7%), myelodysplastic syndrome (30.6% vs. 28.2%), and chronic myelomonocytic leukemia (11.1% vs. 21.1%) (*p* = 0.44).

The proportion of patients with high or very high DRI was comparable between the PTCy and TAC/MTX groups (32.3% vs. 43.6%, *p* = 0.30). Among patients with AML, the incidence of adverse‐risk classification per ELN2022 criteria was similar across both cohorts (71.4% vs. 75%, *p* = 0.66). Notably, the PTCy group exhibited a higher proportion of MRD+ AML cases (25% vs. 7%, *p* = 0.02). On the other hand, the PTCy group was associated with less active disease (11.1%), compared to 29.6% in the TAC/MTX group (*p* = 0.03). Otherwise, fewer patients in the PTCy group had a high hematopoietic cell transplantation comorbidity index (HCT‐CI ≥ 3) (38.9% vs. 56.3%, *p* = 0.09).

The prevalence of *TP53* mutations was comparable between the PTCy and TAC/MTX groups (25% vs. 18.3%, *p* = 0.42). Similarly, no differences in the distribution of cytogenetic abnormalities were found: CK (33.3% vs. 29.6%, *p* = 0.69), CK/MK (16.7% vs. 23.9%, *p* = 0.39), chromosome 7 abnormalities (27.8% vs. 18.3%, *p* = 0.26), and chromosome 17 abnormalities (13.9% vs. 14.1%, *p* = 0.98). The utilization of post‐transplant maintenance therapy for relapse prevention was comparable between PTCy and TAC/MTX (36.1% vs. 31%, *p* = 0.59).

The 2‐yr OS was comparable between the PTCy and TAC/MTX groups (59.2% vs. 63.9%, *p* = 0.67, Figure 1A). There was a trend towards an inferior 2‐year DFS in the PTCy group (37.5% vs. 56.7%, *p* = 0.07, Figure 1B). This trend for 2‐year DFS in the PTCy group was also seen in univariate Cox‐proportional hazard analysis (HR 1.67, 95% CI 0.94–2.97, *p* = 0.08; Table S1). In multivariate analysis, which included *TP53* mutation and CK/MK as other co‐variates, PTCy was associated with an inferior DFS (HR 2.02, 95% CI 1.09–3.73, *p* = 0.03; Table S2). Additionally, PTCy was associated with an increased risk of relapse (2‐year CIR: 50.2*%* vs. 17.3*%, p* < 0.001, Figure 1C, Table S3). Multivariate competing risk analysis, which included *TP53* mutation and CK/MK as co‐variates, further confirmed this association, demonstrating an increased relapse risk with PTCy (HR 4.74, 95% CI 1.97–11.42, *p* < 0.001, Table S4). The 2‐year GRFS was comparable between the PTCy and TAC/MTX groups (28.1% vs. 25.1%, *p* = 0.54). Among patients receiving PTCy, CIR was numerically higher among MRD/MUD compared to haploidentical donors (2‐year CIR: 61.1% vs. 36.1%, *p* = 0.41).

**FIGURE 1:**
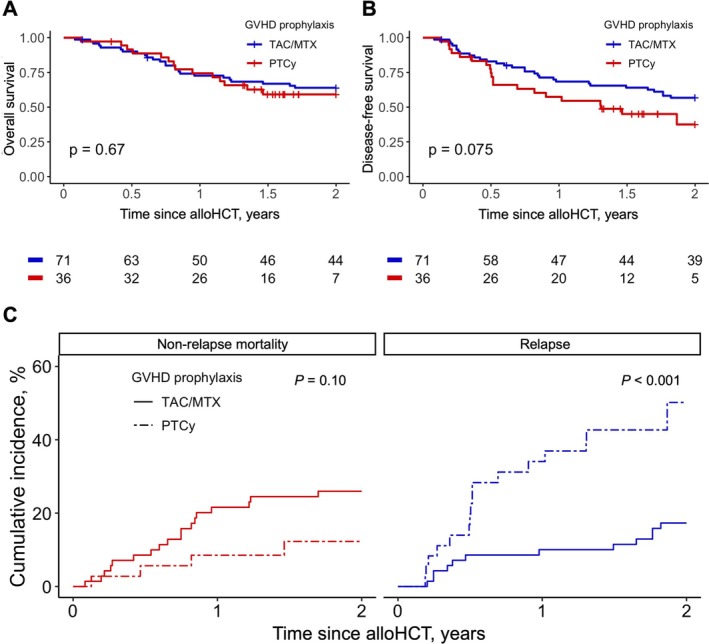
(A). OS after alloHCT stratified by PTCy vs. TAC/MTX, (B). DFS after alloHCT, stratified by PTCy vs. TAC/MTX, (C). Post‐alloHCT NRM and RI stratified by PTCy vs. TAC/MTX.

2

### 2.1 Subgroup Analysis of CIR

In MRD/MUD transplants, PTCy was an independent predictor of relapse (HR 6.7, 95% CI 2.63–17.09, *p* < 0.001; Tables S5 and S6). Competing risk analysis further confirmed this association, showing a significantly higher risk of relapse in PTCy when compared to TAC/MTX (2‐year CIR; 61.1% vs. 16.3%; *p* < 0.001). Similar findings were observed in patients with AML (39.4% vs. 17.1%, *p* = 0.01), myelodysplastic syndrome (66.7% vs. 15%, *p* = 0.01), and secondary AML (47.6% vs. 0.0%, *p* = 0.01). Mutations that were associated with a higher relapse risk in the PTCy group included *TP53* (66.7% vs. 30.7%, *p* = 0.02, Figure S1) and *ASXL1* (37% vs. 4.7%, *p* = 0.03). Among patients with CK/MK, PTCy was associated with an increased risk of relapse compared to TAC/MTX (2‐year CIR: 83.3% vs. 29.4%, *p* = 0.001). Similarly, among patients with chromosome 17 abnormality, PTCy was associated with an increased risk of relapse, in contrast to the TAC/MTX group (2‐year CIR: 100% vs. 30%, *p* < 0.001). Relapse risk was also significant in patients receiving PTCy with high or very high DRI (70% vs. 25%, *p* = 0.004) and intermediate DRI (44.2% vs. 11%, *p* = 0.01). Out of 45 AML patients who had MRD assessment before transplant, 14 (31.1%) had MRD+. Among MRD+ AML, PTCy was associated with a numerically higher relapse rate compared to TAC/MTX (2‐year CIR: 66.7% vs. 40%, *p* = 0.10).

### 2.2 Subgroup Analysis of DFS

Among MRD/MUD, patients receiving PTCy had an inferior 2‐year DFS compared to TAC/MTX (30.6% vs. 58.4%, *p* = 0.05). Similarly, patients receiving PTCy had an inferior DFS among those with CK/MK (16.7% vs. 29.4%, *p* = 0.03), chromosome 17 abnormalities (0% vs. 40%, *p* = 0.004), and *RUNX1* mutations compared to TAC/MTX (37.5% vs. 76.2%, *p* = 0.03).

### 2.3 Study Limitations

Our study has several limitations that should be acknowledged. As a single‐center, retrospective analysis, its generalization may be limited. Additionally, the relatively small sample size reduces the statistical power of subgroup analyses; particularly, it precludes stratifying outcomes by somatic mutations. Furthermore, the follow‐up time for the PTCy group was shorter than the TAC/MTX group. Despite that, the observed higher CIR in the PTCy group emphasizes a significant association between PTCy‐based GvHD prophylaxis and increased relapse risk.

### 2.4 Conclusions

Our study is the first study to stratify the risk of relapse based on GvHD prophylaxis in patients with high‐risk myeloid neoplasms undergoing RIC/NMA alloHCT. Shaffer et al. previously reported an increased relapse risk with PTCy versus calcineurin inhibitor (CNI)‐based prophylaxis in MUD transplants [6]. Similar findings were reported by Kean et al. when PTCy was compared to Abatacept‐based GvHD prophylaxis [7]. We found that, in HR‐MNs, PTCy is associated with a significantly higher risk of relapse compared to TAC/MTX, and this higher relapse risk was observed across all donor types. Notably, despite the inferior DFS and higher relapse rates in the PTCy group, the OS remained comparable, likely due to increased GvHD and NRM in the TAC/MTX cohort. However, the previously reported superior GRFS was lost in patients who received PTCy.

Indeed, further validation of our findings is required in a larger database. Nevertheless, we highlight that a single type of GvHD prophylaxis may not be universally suitable for all patients. Our findings urge the importance of refining GvHD prevention strategies in HR‐MN patients undergoing MRD/MUD alloHCT to optimize relapse prevention and survival outcomes.

## Author Contributions

H.B.A. conceptualized the study. K.H., A.B., and H.B.A. contributed to study design, analyzed the data, and wrote the first draft of the manuscript. A.R.K. and K.H. contributed to data acquisition. J.B., G.B., and R.W. reviewed the manuscript. A.Mat., M.H., A.Man., M.R.L., W.J.H., D.D., and M.V.S. contributed patients and reviewed the manuscript. All authors approve the final version of the manuscript.

## Ethics Statement

The study was approved by the Mayo Clinic institutional review board as a minimal risk study. The study was conducted in accordance with the Helsinki Declaration.

## Conflicts of Interest

The authors declare no conflicts of interest.

## Supporting information


**Data S1:** Supporting Information.

## Data Availability

The dataset generated during and/or analyzed during the current study are not publicly available due to patient confidentiality and privacy restrictions. However, they are available from the corresponding author upon a reasonable request and subject to institutional approval.
