# Supplementary material for: Risk of Relapse Post Reduced Intensity Conditioning Allogeneic Stem Cell Transplant in Patients With High‐Risk Myeloid Neoplasms Based on GvHD Prophylaxis: PTCy Vs. TAC/MTX
Source: Am J Hematol. 2025 Sep 4;100(11):2134–9. doi: 10.1002/ajh.70059 (PMC12516666; doi:10.1002/ajh.70059)
Supplement: Supplementary file 1 — Data S1: Supporting Information. [file AJH-100-2134-s001.docx]

**Supplemental Tables:**

| **Variable** | **HR** | **Lower CI** | **Upper CI** | ***P*** |
| --- | --- | --- | --- | --- |
| Disease |  |  |  |  |
| CMML (vs. AML) | 1.15 | 0.53 | 2.48 | 0.73 |
| MDS (vs. AML) | 1.53 | 0.83 | 2.85 | 0.18 |
| FLT3 | 1.24 | 0.53 | 2.91 | 0.62 |
| KRAS | 0.45 | 0.06 | 3.29 | 0.43 |
| WT1 | 0.24 | 0.03 | 1.71 | 0.15 |
| TP53 | 2.25 | 1.23 | 4.12 | **0.01** |
| NRAS | 1.29 | 0.58 | 2.87 | 0.54 |
| Number of adverse-risk mutations | 0.74 | 0.40 | 1.36 | 0.34 |
| Multi-hit TP53 | 0.87 | 0.32 | 2.34 | 0.78 |
| Monosomal karyotype | 0.58 | 0.08 | 4.22 | 0.59 |
| Complex karyotype | 1.49 | 0.84 | 2.65 | 0.17 |
| Complex/Monosomal karyotype | 2.39 | 1.33 | 4.29 | **<0.01** |
| Chromosome 17 abnormality | 1.93 | 0.99 | 3.77 | **0.06** |
| Chromosome 7 abnormality | 1.75 | 0.93 | 3.31 | **0.08** |
| sAML | 0.95 | 0.39 | 2.31 | 0.90 |
| tMN | 1.07 | 0.38 | 3.01 | 0.90 |
| CR at transplant | 1.10 | 0.56 | 2.15 | 0.78 |
| High/Very High DRI | 1.94 | 1.04 | 3.62 | **0.04** |
| High HCT-CI | 1.30 | 0.74 | 2.27 | 0.36 |
| Major or Bidirectional ABO Mismatch | 1.83 | 0.82 | 4.07 | 0.14 |
| PTCY | 1.67 | 0.94 | 2.97 | **0.08** |
| Age at transplant 65 or above | 1.13 | 0.64 | 2.00 | 0.67 |
| TP53 mutation by hit status (vs. no TP53) |  |  |  |  |
| Single hit | 2.38 | 1.09 | 5.19 | **0.03** |
| Multi-hit | 2.14 | 0.98 | 4.65 | **0.06** |
| Disease |  |  |  |  |
| Primary AML (vs. no AML) | 0.73 | 0.40 | 1.37 | 0.33 |
| Secondary AML (vs. no AML) | 0.70 | 0.31 | 1.62 | 0.41 |
| Maintenance | 0.94 | 0.50 | 1.77 | 0.85 |
| CMML: Chronic myelomonocytic leukemia; MDS: Myelodysplastic syndrome; AML: Acute myeloid leukemia; sAML: Secondary acute myeloid leukemia; tMN: Therapy-related myeloid neoplasm; CR: Complete remission; DRI: Disease risk index; HCT-CI: Hematopoietic cell transplant specific comorbidity index; PTCY: Post-transplant cyclophosphamide | | | | |

**Supplemental Table 1:** Univariate Analysis (UVA) for Disease-Free Survival 2 years after alloHCT.

| **Variable** | **HR** | **Lower CI** | **Upper CI** | ***P*** |
| --- | --- | --- | --- | --- |
| TP53 | 1.29 | 0.58 | 2.86 | 0.53 |
| Complex/Monosomal karyotype | 2.45 | 1.09 | 5.48 | **0.03** |
| PTCY | 2.02 | 1.09 | 3.73 | **0.03** |
| PTCY: Post-transplant cyclophosphamide | | | | |

**Supplemental Table 2:** Multivariate analysis (MVA) for Disease-Free Survival 2 years after alloHCT.

| **Variable** | **HR** | **Lower CI** | **Upper CI** | ***P*** |
| --- | --- | --- | --- | --- |
| FLT3 | 0.95 | 0.30 | 2.96 | 0.92 |
| KRAS | 0.94 | 0.12 | 7.14 | 0.95 |
| WT1 | NA | NA | NA | NA |
| TP53 | 2.41 | 1.13 | 5.15 | **0.02** |
| NRAS | 1.60 | 0.62 | 4.15 | 0.34 |
| Multi-hit TP53 | 1.46 | 0.43 | 4.96 | 0.54 |
| Complex Karyotype | 1.88 | 0.89 | 3.97 | **0.10** |
| Complex/Monosomal karyotype | 2.39 | 1.10 | 5.18 | **0.03** |
| Chromosome 17 abnormality | 2.77 | 1.26 | 6.09 | **0.01** |
| Chromosome 7 abnormality | 1.79 | 0.77 | 4.15 | 0.18 |
| sAML | 0.52 | 0.14 | 1.91 | 0.32 |
| tMN | 0.87 | 0.20 | 3.74 | 0.85 |
| CR at transplant | 1.50 | 0.56 | 4.03 | 0.42 |
| High/Very High DRI | 2.25 | 1.00 | 5.06 | **0.05** |
| PTCY | 3.67 | 1.75 | 7.70 | **<0.01** |
| sAML: Secondary acute myeloid leukemia; tMN: Therapy related myeloid neoplasm; CR: Complete remission; DRI: Disease risk index; PTCY: Post-transplant cyclophosphamide | | | | |

**Supplemental Table 3:** Univariate Analysis (UVA) for Competing Risk Analysis 2 years after alloHCT.

**Supplemental Table 4:** Multivariate analysis (MVA) for competing risk analysis 2 years after alloHCT.

| **Variable** | **HR** | **Lower CI** | **Upper CI** | ***P*** |
| --- | --- | --- | --- | --- |
| TP53 | 1.25 | 0.44 | 3.59 | 0.68 |
| Complex/Monosomal karyotype | 3.12 | 0.97 | 10.03 | **0.06** |
| PTCY | 4.74 | 1.97 | 11.42 | **<0.01** |
| PTCY: Post-transplant cyclophosphamide | | | | |

**Supplemental Table 5:** Univariate Analysis (UVA) for Competing Risk Analysis 2 years after alloHCT in MRD/MUD transplants.

| **Variable** | **HR** | **Lower CI** | **Upper CI** | ***P*** |
| --- | --- | --- | --- | --- |
| FLT3 | 1.14 | 0.36 | 3.63 | 0.83 |
| KRAS | 1.01 | 0.13 | 7.70 | 0.99 |
| WT1 | NA | NA | NA | NA |
| TP53 | 3.12 | 1.38 | 7.04 | **<0.01** |
| NRAS | 1.73 | 0.63 | 4.80 | 0.29 |
| Multi-hit TP53 | 1.31 | 0.38 | 4.45 | 0.67 |
| Complex karyotype | 2.20 | 0.98 | 4.93 | **0.06** |
| Complex/Monosomal karyotype | 2.55 | 1.11 | 5.87 | **0.03** |
| Chromosome 17 abnormality | 2.30 | 0.95 | 5.57 | **0.06** |
| Chromosome 7 abnormality | 1.71 | 0.65 | 4.48 | 0.28 |
| sAML | 0.20 | 0.03 | 1.66 | 0.14 |
| tMN | 0.49 | 0.06 | 4.05 | 0.51 |
| CR at transplant | 1.70 | 0.57 | 5.04 | 0.34 |
| High/Very high DRI | 1.60 | 0.66 | 3.90 | 0.30 |
| PTCy | 4.86 | 2.17 | 10.92 | **<0.01** |
| sAML: Secondary acute myeloid leukemia; tMN: Therapy related myeloid neoplasm; CR: Complete remission; DRI: Disease risk index; PTCY: Post-transplant cyclophosphamide | | | | |

**Supplemental Table 6:** Multivariate analysis (MVA) for competing risk analysis 2 years after alloHCT in MRD/MUD transplants.

| **Variable** | **HR** | **Lower CI** | **Upper CI** | ***P*** |
| --- | --- | --- | --- | --- |
| TP53 | 1.65 | 0.66 | 4.08 | 0.28 |
| Complex/Monosomal karyotype | 3.16 | 1.18 | 8.48 | **0.02** |
| PTCY | 6.7 | 2.63 | 17.09 | **<0.01** |
| PTCY: Post-transplant cyclophosphamide | | | | |

**Supplemental Figures:**

**Figure S1:**

**
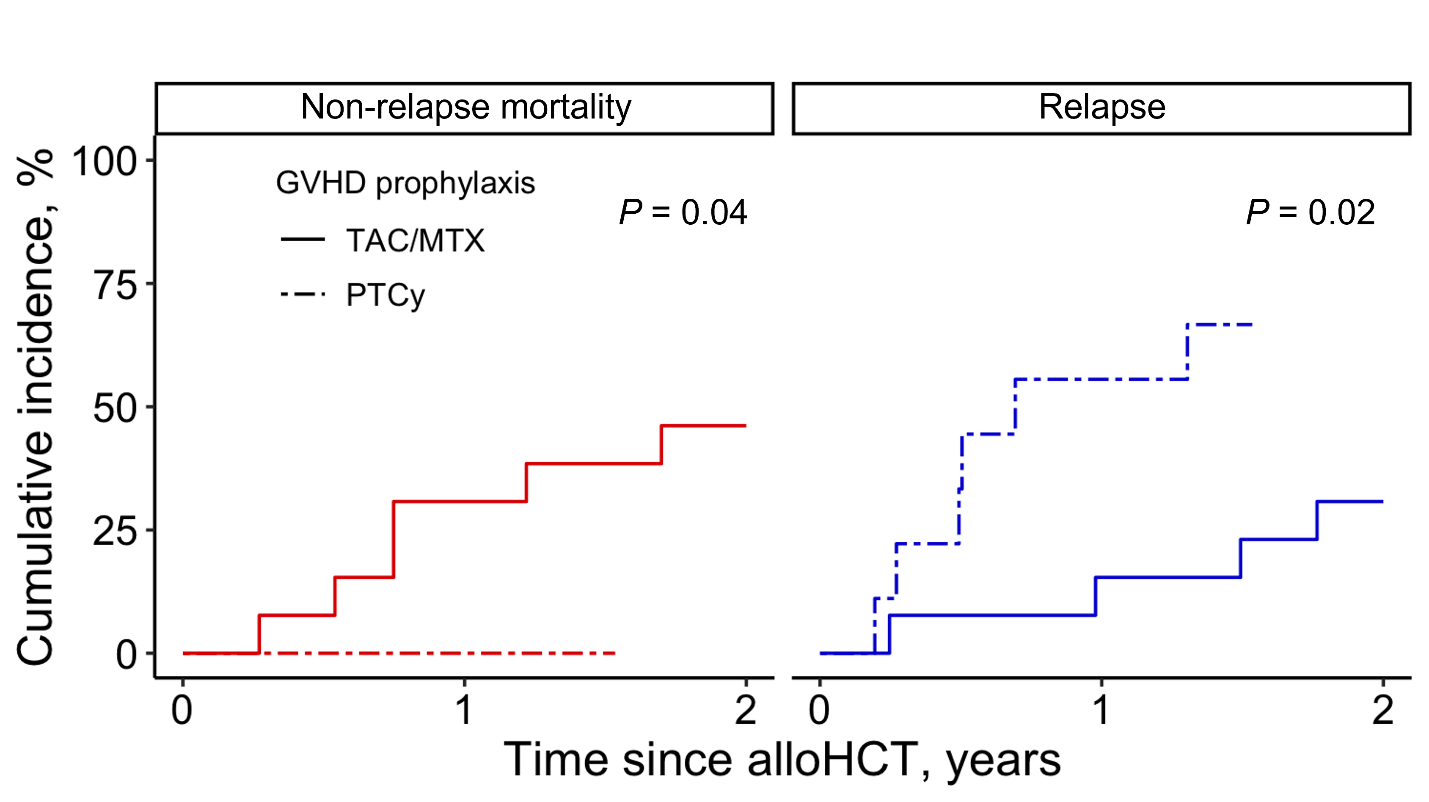
**

**Figure S1**: Post-alloHCT NRM and RI stratified by PTCy *vs.* TAC/MTX in patients with *TP53* mutations.
